# 627. β-lactam-β-lactamase Inhibitor Combinations Show a High Degree of Cross Resistance in Gram-negative Bacteria But Not to Cefiderocol; Results from the SENTRY Antimicrobial Surveillance Program (2020-2024)

**DOI:** 10.1093/ofid/ofaf695.194

**Published:** 2026-01-11

**Authors:** Boudewijn L DeJonge, Sean T Nguyen, Joshua Maher, Rodrigo E Mendes, Christopher M Longshaw, Hidenori Yamashiro, Yoshinori Yamano

**Affiliations:** Shionogi Inc., Florham Park, NJ; Shionogi Inc., Florham Park, NJ; Element Materials Technology/Jones Microbiology Institute, North Liberty, Iowa; Element Iowa City (JMI Laboratories), North Liberty, IA; Shionogi B.V., London, England, United Kingdom; Shionogi & Co., Ltd., Toyonaka, Osaka, Japan; Shionogi & Co., Ltd., Toyonaka, Osaka, Japan

## Abstract

**Background:**

β-lactam-β-lactamase inhibitor combinations (BL-BLICs) such as ceftazidime-avibactam (CZA), meropenem-vaborbactam (MVB), ceftolozane-tazobactam (C/T) and imipenem-relebactam (IMR) are used to treat infections caused by carbapenem-resistant Enterobacterales and/or *P. aeruginosa*. Resistance for these agents was assessed for isolates collected in a five year surveillance program and the degree of cross resistance amongst BL-BLICs and the siderophore conjugated cephalosporin cefiderocol (FDC) was determined.

**Table 1.**
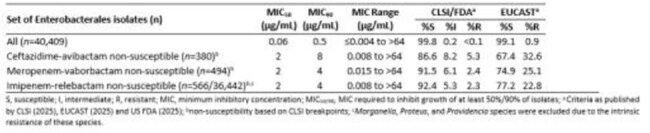
Activity of cefiderocol against ceftazidime-avibactam-, meropenem-vaborbactam-, and imipenem-relebactam-non-susceptible Enterobacterales isolates collected from European and US hospitals as part of the SENTRY surveillance program (2020-2024).

**Table 2.**
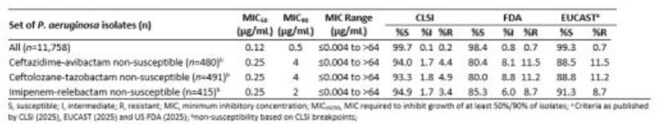
Activity of cefiderocol against ceftazidime-avibactam-, meropenem-vaborbactam-, and imipenem-relebactam-non-susceptible P. aeruginosa isolates collected from European and US hospitals as part of the SENTRY surveillance program (2020-2024).

**Methods:**

40,408 Enterobacterales and 11,757 *P. aeruginosa* were collected during 2020-2024 in Europe and the USA as part of the SENTRY Antimicrobial Surveillance Program. Minimum inhibitory concentrations were determined according to CLSI procedures with broth microdilution using cation-adjusted Mueller-Hinton broth (CAMHB) for BL-BLICs and iron-depleted CAMHB for FDC. Susceptibility was assessed according to 2025 CLSI, FDA, and EUCAST breakpoints. Non-susceptibility (NS) to BL-BLICs was assessed with CLSI breakpoints.

**Figure 1:**
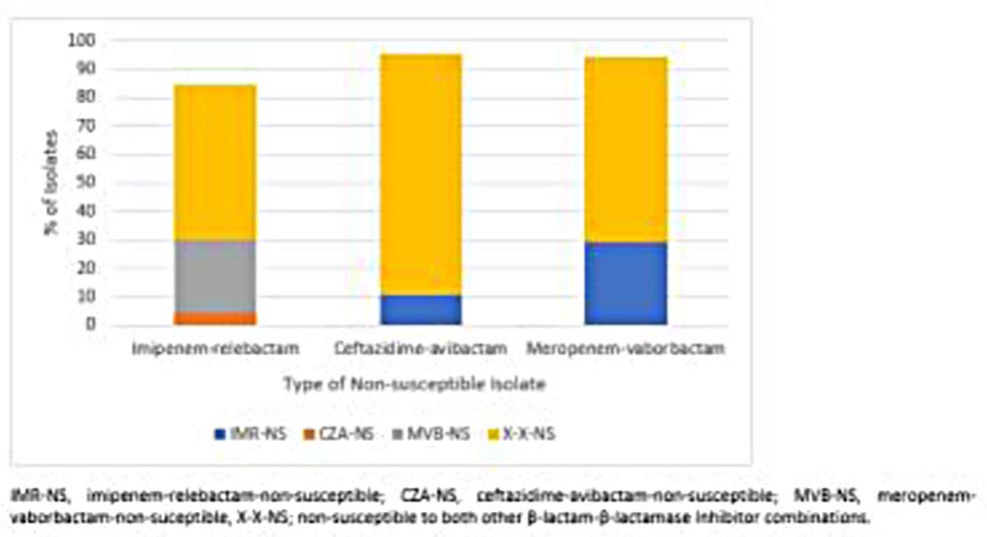
Degree of cross resistance amongst β-lactam-β-lactamase inhibitor combinations for ceftazidime-avibactam-, meropenem-vaborbactam-, and imipenem-relebactam-non-susceptible Enterobacterales isolates collected from European and US hospitals as part of the SENTRY surveillance program (2020-2024).

**Figure 2:**
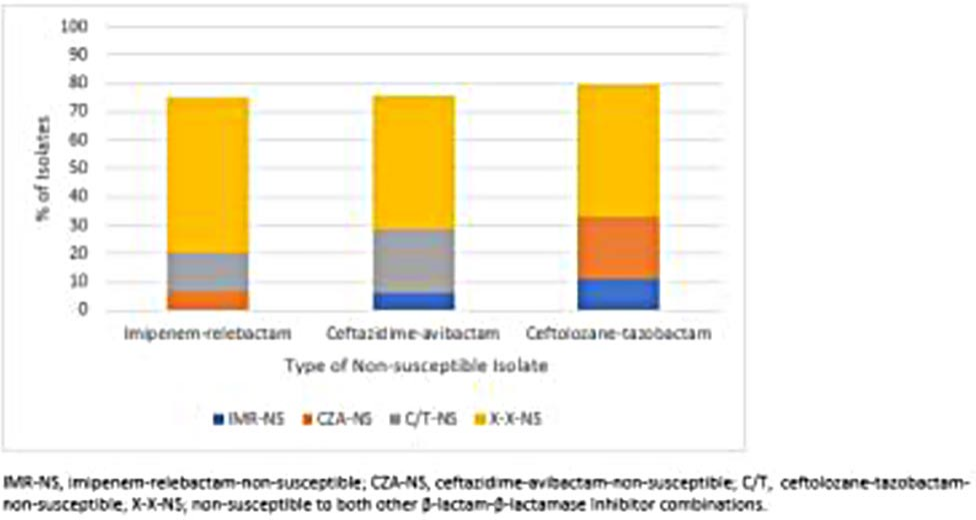
Degree of cross resistance amongst β-lactam-β-lactamase inhibitor combinations for ceftazidime-avibactam-, ceftolozane-tazobactam-, and imipenem-relebactam-non-susceptible P. aeruginosa isolates collected from European and US hospitals as part of the SENTRY surveillance program (2020-2024).

**Results:**

0.9% (380/40,408), 1.2% (494/40,408), and 1.6% (566/36,441) of Enterobacterales were NS to CZA, MVB, and IMR, respectively. The majority of these isolates were also NS to other BL-BLICs; 84.8% of IMR-NS isolates were NS to one or both other BL-BLICs and these percentages were 95.5% for CZA-NS and 94.3% for MVB-NS isolates (Fig. 1). In contrast, 86.6%, 91.5%, and 92.4% of the CZA-NS, MVB-NS, and IMR-NS isolates remained susceptible to FDC, respectively, according to CLSI breakpoints (Table 1). For *P. aeruginosa*, 4.1% (480/11,757), 4.2% (491/11,757), and 3.5% (415/11,757) were CZA-, C/T-, and IMR-NS, respectively. 74.9% of IMR-NS isolates were also NS to one or both other BL-BLICs , and these percentages were 76.8% for CZA-NS and 79.4%% for I/R-NS isolates (Fig. 2). However, 94.0%, 93.3%, and 94.9% of the CZA-NS, C/T-NS, and IMR-NS isolates remained susceptible to FDC, respectively, according to CLSI breakpoints (Table 2).

**Conclusion:**

BL-BLICs showed a high degree of cross resistance in Enterobacterales and *P. aeruginosa*, but FDC remained active against these highly resistant isolates. FDC should be considered as a treatment option when pathogens NS to any BL-BLICs are encountered.

**Disclosures:**

All Authors: No reported disclosures

